# The influence of fasudil on renal proximal tubular cell epithelial–mesenchymal transition induced by parathormone

**DOI:** 10.1080/0886022X.2017.1349677

**Published:** 2017-07-25

**Authors:** Ziqing Gao, Weiping Zhu, Hua Zhang, Zhonghe Li, Tongxia Cui

**Affiliations:** a Department of Ultrasound, the Fifth Affiliated Hospital of Sun Yat-Sen University, Zhuhai, China;; b Department of Nephrology, the Fifth Affiliated Hospital of Sun Yat-Sen University, Zhuhai, China

**Keywords:** Renal proximal tubular epithelial cells (HK-2), fasudil, parathormone, epithelial–mesenchymal transition (EMT), renal fibrosis

## Abstract

**Background:** Renal fibrosis is a common pathway through which a variety of chronic kidney diseases progress to end-stage renal disease. Epithelial–mesenchymal transition (EMT) of renal proximal tubular cells is one of the most important factors in renal fibrosis. This study investigates if fasudil could influence EMT of renal proximal tubular cells.

**Methods:** HK-2 cells in passage 3–4 were used for all experiments. The cells were divided into five groups and treated with different concentrations of PTH and then observe cellular morphological changes at 0, 24 and 48 h using an inverted microscope and investigate the expression of the epithelial cell marker E-cadherin and the renal fibroblast marker α-smooth muscle actin (α-SMA).

**Results:** PTH significantly induced EMT, fasudil-inhibited EMT induced by PTH to different degrees, and the inhibitory effect of fasudil was most pronounced at 20 μmol/L.

**Conclusion:** Monitoring PTH levels, early prevention and control of hyperparathyroidism and reducing the concentration of PTH are important means to improve prognosis and delay the progression of chronic kidney disease. Fasudil can restrain EMT induced by PTH; this conclusion provides experimental data for the application of fasudil in the clinical prevention and treatment of renal fibrosis.

## Introduction

Previous studies have demonstrated that the main pathological changes of end-stage renal disease are renal glomerular sclerosis and renal interstitium fibrosis. There is a high correlation with renal fibrosis and kidney malfunction, as the renal tubule is the hub connecting the glomerular and renal interstitium. Epithelial–mesenchymal transition (EMT) is one of the most important factors in renal fibrosis. In 1995, Strutz et al. [1–3] first proposed that renal tubular epithelial cells could be converted into mesenchymal cells through EMT. During the transdifferentiation process, renal tubular cells were found to have lost epithelial markers, such as E-cadherin and keratin, but gained expression of renal interstitium markers, such as α-smooth muscle actin (α-SMA) and vimentin. In 2002, Iwano M et al. [4] found that 14–15% of the fibrotic cells were renal fibroblasts from the bone marrow and 36% came from EMT, proving that EMT is the main source of renal fibroblasts. Therefore, renal tubular epithelial cells play an important role in the progression of renal fibrosis. Studies regarding the transformation of renal tubular epithelial cell have high scientific and clinical value for the prevention and treatment of renal fibrosis and delaying the progression of kidney disease and improving prognosis.

Recently, it was reported that parathormone (PTH) could promote the expression of α-SMA and TGF-β1 in renal tubular epithelial cells. α-SMA and TGF-β1 expression reached peak values when epithelial cells were cultured with 10^−10 ^mol/L PTH for 48 h, and with rising PTH concentrations α-SMA and TGF-β1 expression declined slightly but were still greater than the control group. PTH also increased the percentage of α-SMA-positive epithelial cells and the expression of α-SMA protein. This discovery suggests that PTH can increase the transdifferentiation potential of renal tubule epithelial cells [5].

Fasudil is a widely used inhibitor of the Rho/Rho kinase (ROCK)-signaling pathway, and in previous studies has been shown to dilate vessels, improve microcirculation in brain tissues, reduce endothelial cell tension, antagonize inflammatory cytokines, protect against neural apoptosis and facilitate neuro regeneration [6–8]. Fasudil is also the earliest Rho/ROCK pathway inhibitor approved for clinical use. It is widely used in *in vivo* and *in vitro* experiments, primarily for curing and preventing ischemic cerebrovascular disease [9]. The therapeutic effect of fasudil on diabetic nephropathy has been verified in animal experiments; it can reduce proteinuria, alleviate glomerular mesangial matrix accumulation and retard the development of glomerular sclerosis and renal interstitial fibrosis [10–14].

The study by Rodrigues-Diez et al. showed that fasudil was able to block Rho/ROCK signaling and inhibited EMT of HK-2 cells induced by Angiotensin II [15]. Gu et al. also found that fasudil could inhibit the Rho/ROCK-signaling pathway in human mesangial cells activated by high sugar, alleviating the inflammation and fibrosis of mesangial cell [16]. Together, these data demonstrate that the Rho/ROCK signaling pathway plays an important role in EMT.

Recent studies have also confirmed that a Rho/ROCK-signaling pathway inhibitor could alleviate EMT in the kidney induced by high sugar, prevent renal fibrosis induced by unilateral ureteral obstruction and diabetic nephropathy in rats. However, whether a Rho/ROCK inhibitor can inhibit EMT induced by PTH has not been reported.

## Materials and methods

### Materials

The human renal tubular epithelial cell line HK-2 was obtained from ATCC (Manassas, VA). Dulbecco’s modified Eagle’s medium (DMEM) and penicillin/streptomycin (10,000 IU/mL and 10 mg/mL) were purchased from Gibco Inc. (Grand Island, NY). Fetal bovine serum (FBS) was obtained from Biological Industries (Beit Haemek, Israel). Human PTH (1–34) was acquired from Sigma Inc. (St. Louis, MO). Fasudil was provided by Tianjin Chase Sun Co. (Tianjin, China) and human epithelial growth factor was purchased from Peprotech Inc. (Rocky Hill, NJ). Anti-Rabbit IgG-FITC antibody, E-cadherin antibody and α-SMA antibody were purchased from Santa Cruz Biotechnology Inc. (Santa Cruz, CA). Primers were designed and synthesized by Life Technologies (Invitrogen, Guangzhou, China). The reverse transcription reagent kit and the real-time polymerase chain reaction (PCR) reagents were purchased from TaKaRa Bio Inc. (Dalian, China).

### Experimental design

The original generation of renal tubular epithelial cells were cultured in DMEM containing 10% (v/v) fetal bovine serum, 1% (v/v) epithelial growth factor (1 ng/mL), 100 U/mL penicillin and 100 μg/mL streptomycin. Cells were incubated at 37 °C in an atmosphere of 95% air and 5% carbon dioxide until they reached 90% confluence, then were harvested in trypsin/EDTA, counted with a hemocytometer, reseeded at 104 cells/cm^2^, and third-generation cells were used in experiments. For all experiments, cells were divided into five groups:

1. Control group (C): PTH (0 mol/L)+fasudil (0 μmol/L);
2. Experimental group 1 (T1): PTH (10^−10^ mol/L)+fasudil (0 μmol/L);
3. Experimental group 2 (T2): PTH (10^−10^ mol/L)+fasudil (10 μmol/L);
4. Experimental group 3 (T3): PTH (10^−10^ mol/L)+fasudil (20 μmol/L);
5. Experimental group 4 (T4): PTH (10^−10^ mol/L)+fasudil (30 μmol/L).

Different concentrations of fasudil (0, 10, 20 and 30 μmol/L) and 10^−10 ^mol/L PTH were added to the culture medium of the renal tubular epithelial cells for 48 h. In the control group, the renal tubular epithelial cells were cultured in medium containing vehicle lacking PTH and fasudil.

### Morphological assessment

Human renal tubular epithelial cells were seeded in culture bottles at 1 × 10^4^ cells/mL. Cells were incubated at 37 °C in an atmosphere of 95% air and 5% carbon dioxide until they reached 80% confluence, then the cells were cultured in serum-free DMEM for 12 h to synchronize growth. The appropriate PTH and fasudil concentrations were added to the cells for 48 h, and morphological changes were observed and documented using an inverted microscope at 0, 24 and 48 h.

### Real-time PCR

After 48-h culture, total RNA was isolated from renal tubular epithelial cells with Trizol reagent (Invitrogen, Carlsbad, CA). For real-time PCR analysis, cDNAs were synthesized from 1 μg of total RNA using reverse transcriptase and oligo dT primers in a 10 μL volume, and the reaction mixture was adjusted to 50 μL with Tris/EDTA buffer for PCR analysis. The cDNA amplification reaction mixture was initially incubated at 95 °C for 30 s to denature DNA; amplification was performed for 40 cycles of 95 °C for 5 s and 60 °C for 34 s. Real-time PCR was performed under the following conditions: 95 °C for 2 min, then 40 cycles of 95 °C, 35 s; 60 °C, 1 min; 72 °C, 2 min. The specificity of the PCR products was verified by melting curve analysis. Data were normalized using actin as a housekeeping gene for an endogenous control. The following primer sets were used: E-cadherin: 5′-CCGGGACAACGTTTATTACTAT, CATAGTCAAACACGAGCAGAGAAT-3′; α-SMA: 5′-CTTGAGAAGAGTTACGAGTTGC, GATGCTGTTGTAGGTGGTTTC-3′; Actin: 5′-CACCCAGCACAATGAAGATCAAGAT, CCAGTTTTTAAATCCTGAGTCAAGC-3′. The relative expression ratio was calculated according to the 2^−ΔΔCT^ method.

### Western blot analysis

After being cultured in dishes for 48 h as described above, the renal tubular epithelial cells were rinsed three times with Tris-buffered saline, and then, RIPA lysis buffer was added to the culture dishes and the reagents were fully mixed with the cells by repeatedly shaking. The cells were then scraped down with a cell scraper and collected. Cell lysate (30 μg) was electrophoresed on 12% polyacrylamide-SDS gels, and proteins were transferred to a nitrocellulose membrane and hybridized with the following donkey anti-mouse antibodies (0.2 μg/mL), donkey anti-rabbit antibodies (0.2 μg/mL) as internal control, followed by 1 μg/mL anti-donkey immunoglobulin G-horseradish peroxide conjugate (Santa Cruz Biotechnology Inc., Santa Cruz, CA). Bands were scanned and the intensity was measured using a digital gel electrophoresis image processing and analysis system (Tanon, Shanghai, China).

### Immunofluorescence

Renal tubular epithelial cells were seeded at 1 × 10^4^ cells/cm^2^ in six-well culture plates, which were covered with poly-L-lysine-Prep slides. Cells were cultured in basic medium until reaching 80% confluence, then were cultured for 48 h in different concentrations of PTH and fasudil. After culture, cells were fixed with 4% paraformaldehyde for 30 min, permeabilized with 0.2% Triton X-100 for 10 min and blocked with 5% bovine serum albumin at room temperature for 30 min. Next, the cells were incubated with anti-α-SMA antibody (1:50) and anti-E-cadherin antibody (1:200) at 4 °C overnight. After washing with phosphate-buffered saline, the slides were incubated with secondary antibody and the slides were sealed after dyeing the nucleus with DAPI. The cells were observed and documented using laser-scanning confocal microscopy.

### Statistical analysis

Data were statistically analyzed using SPSS v21.0 software (SPSS Inc., Chicago, IL). The results are represented as mean ± standard deviation (SD). mRNA expression data were analyzed using the 2^−ΔΔCT^ method. Differences among groups were compared by one-factor analysis of variance. Sample means between two groups were compared with the LSD test. *p* < .05 was considered statistically significant.

## Results

### Cell morphology

**Figure 1.**
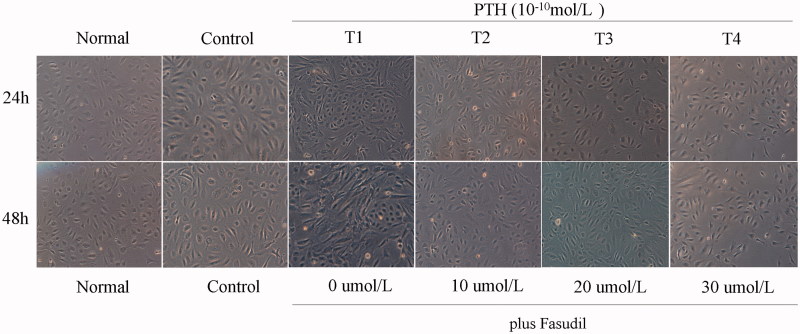
The impact of fasudil on cell morphology. HK-2 cells were treated with 0, 10, 20 and 30 ng/mL Fasudil in the presence of 10^−10^ mol/L PTH for 48 h. Cells were observed with an inverted microscope at 100× magnification.

Renal tubular epithelial cells in the control group were round or oval and arranged as paving stones, as observed under an inverted microscope. Cells in the experimental groups were stretched into long fusiform or irregularly-shaped architecture after stimulation with PTH. Intercellular connections were loose and arranged in parallel stripes. The morphology of the cells changed more dramatically as the PTH stimulation time in T1 was extended. As the concentration of fasudil increased, evidence of EMT gradually reduced in groups T2 and T3. The number of cells undergoing EMT in group T4 increased slightly compared with group T3 but was still less than group T1 (Figure 1).

### Changes in E-cadherin and α-SMA mRNA expression

**Figure 2.**
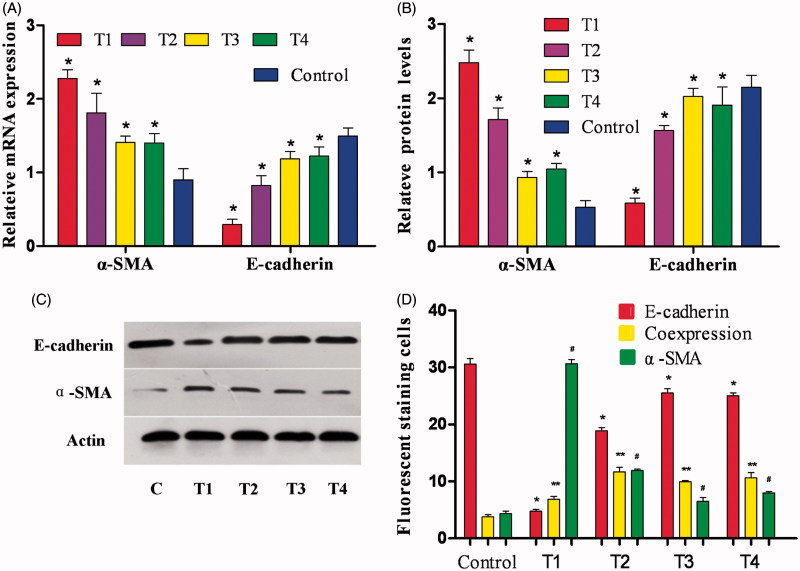
Effect of fasudil on HK-2 cells. (A) HK-2 cells were cultured in each group as discussed in the Methods section. RT-PCR was performed and the expression of E-cadherin and α-SMA was normalized to actin. Results are from three independent experiments performed in triplicate and are displayed as the relative expression normalized to the control samples. Values are the means with S.E.M. shown by vertical bars. *p < .05. (B) Densitometric analysis for western blots. Values are the means from triplicate determinations with the S.E.M. shown by vertical bars. *p < .05. (C) Western blot for E-cadherin and α-SMA using HK-2 cells treated as aforementioned. (D) The number of positive-stained cells by immunofluorescence is shown by vertical bars. *, ** and #*p* < .05.

Compared with the control group, E-cadherin mRNA expression decreased significantly in each group treated with PTH, and α-SMA mRNA expression increased, especially in group T1 (*p* < .05). Compared with groups T3 and T4, there were no statistically significant differences in α-SMA mRNA levels (*p* > .05). In groups T1–T3, E-cadherin mRNA expression increased with increasing concentrations of fasudil, and α-SMA mRNA was gradually reduced. E-Cadherin expression peaked in group T3 and fell slightly in group T4, but there were no statistically significant differences between groups T3 and T4; the difference between group T1 and the other groups did show statistical significance (*p* < .05) (Figure 2(A)).

### Changes in E-cadherin and α-SMA protein expression

Compared with the control group, E-cadherin protein expression decreased significantly in each group treated with PTH, and α-SMA protein expression increased, especially in group T1 (*p* < .05). In groups T1–T4, E-cadherin protein expression increased with increasing concentration of fasudil, and α-SMA protein expression decreased gradually. E-cadherin protein expression peaked in group T3 and fell slightly in group T4, but there were no statistically significant differences between groups T3 and T4. However, the difference between group T1 and the other groups did show statistical significance (*p* < .05) (Figure 2(B,C)).

### Changes in immunofluorescence

**Figure 3.**
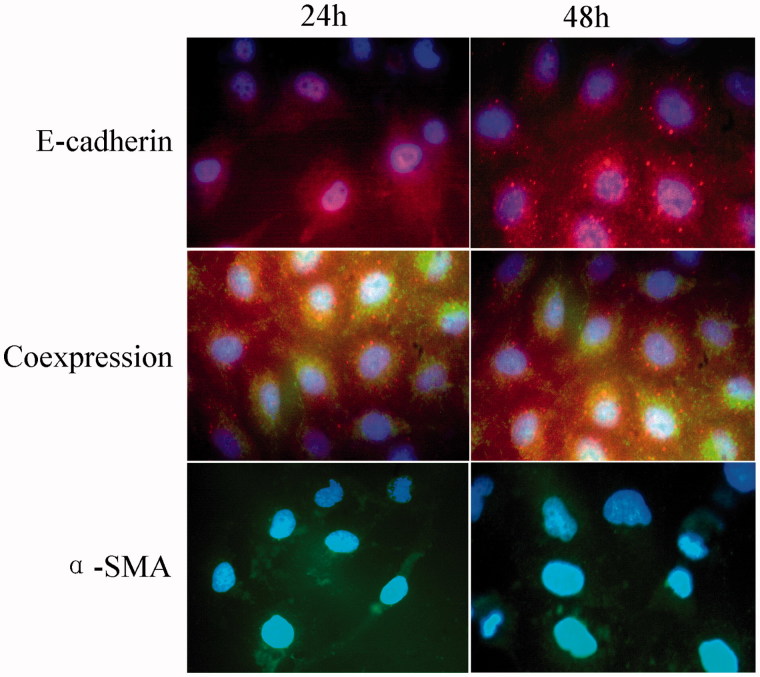
Effect of fasudil on cell immunofluorescence. Immunofluorescence staining of HK-2 cells captured by inverted fluorescence microscope at 100× magnification. The number of cells from 10 random fields for each group was calculated. Data are presented as the mean ± S.E.M. *p < .05 versus control. Red: E-cadherin expression; Green: α-SMA expression; Blue: nuclei (DAPI). Cells coexpressing α-SMA and E-cadherin are yellow. Results are from three experiments performed in triplicate.

Under the fluorescence microscope, immunoreactive cells for the epithelial markers E-cadherin were red, and immunoreactive cells for the mesenchymal marker α-SMA were green; the cell nuclei, stained with DAPI, were blue. In the T1–T4 experimental groups, some cells were green some were red and others showed both red and green fluorescence. These data suggest that both epithelial and mesenchymal markers were expressed in the renal tubular epithelial cells. Compared with the control group, the number of α-SMA-positive cells was increased significantly, and the number of E-cadherin-positive cells decreased significantly in each treatment group. Comparing groups T2–T4 with group T1 demonstrated that fasudil intervention caused an increase in the number of E-cadherin-positive cells and a corollary decrease in the number of α-SMA-positive cells; these differences had statistical significance (Figure 2(D) and 3).

## Discussion

Recently, many doctors have come to the conclusion that PTH expedites the progression of renal fibrosis by stimulating renal tubular epithelial cells to secrete fibronectin and TGF-β1. In the early stages of chronic kidney disease (CKD), serum PTH levels have already increased significantly in most patients [17]. During the course of CKD, 1,25-(OH)_2_D_3_ deficiency, calcium phosphorus metabolism disorders, secondary hyperparathyroidism and serum calcium concentration decreases further stimulate parathyroid secretion of more PTH. The hypothesis that PTH is one of the toxins in the body associated with CKD has now been confirmed. PTH causes soft tissue and cardiovascular calcification, so decreasing PTH levels is essential to delay the progression of CKD. In previous studies, 10^−10 ^mol/L PTH was used in culture with HK-2 cells, and α-SMA mRNA and protein levels were found to peak after 48 h [18,19]. In our study, we observed morphological changes in HK-2 cells after 24-h PTH treatment. The cells were stretched into long fusiform or irregular shapes and intercellular connections were lost. These changes were more obvious after 48 h. The most obvious changes were observed in the T1 group; in immunofluorescence assays, α-SMA-positive cells were increased whereas E-cadherin-positive cells were significantly decreased. This result indicated that renal tubular epithelial cells were induced to transform into renal interstitial cells in the presence of high serum concentration of PTH. This discovery was also supported by western blot and RT-PCR results. Based on the above results, we conclude that PTH induces the transition of human renal tubular epithelial cells into epithelial–mesenchymal cells and this effect can be blocked by fasudil.

The majority of patients with CKD have atypical serum calcium levels and phosphorus metabolism disorders along with decreased renal function. Long-term calcium and phosphorus metabolism disorders lead to hyperparathyroidism, and bone and mineral metabolism disorders. When kidney function is below a certain threshold, low calcium and high phosphorus appear in clinical examinations. High phosphorus levels can directly stimulate the parathyroid gland and indirectly stimulate the secretion of PTH by lowering serum calcium ion concentrations. In the early stages of CKD, most patients already have elevated PTH levels. Therefore, closely monitoring calcium and phosphorus levels, undergoing preventative treatment for hyperparathyroidism, and decreasing serum PTH levels, are essential ways to delay the progression of CKD and improve prognosis.

Recently, the Rho/ROCK signaling pathway has attracted attention as a therapeutic target because it plays important roles in chronic inflammation and fibrosis in variety of organs such as the liver [20] and lungs [21]. But studies regarding the role of the Rho/ROCK-signaling pathway in renal fibrosis have been poor. The Rho/ROCK-signaling pathway is widely expressed throughout the human body. ROCK, also called Rho kinase-related kinases or Rho-associated kinase, is primarily cytoplasmic and belongs to the family of serine/threonine kinases [22]. The Rho family of GTPases is composed of RhoA, RhoB and RhoC and regulates the cytoskeleton, playing essential roles in cell polarity, division, proliferation, apoptosis and tumor cell metastasis [23,24]. Patel et al. [25] found that ROCK activation was a phenotype of renal fibrosis caused by high glucose levels. Koshikawa et al. have confirmed fasudil to be a ROCK-specific inhibitor [26]. After the application of fasudil, ROCK mRNA expression significantly decreased in animal experiments [27]. Fasudil effectively blocks ROCK signaling and is the most commonly used Rho/ROCK pathway inhibitor in clinical settings.

As a selective Rho-kinase inhibitor, fasudil has recently been shown to improve renal damage resulting from unilateral ureteral ligation, hypertensive glomerulosclerosis and subtotal nephrectomy [28,29]. In mice induced with unilateral ureteral obstruction, fasudil suppressed the transformation of renal intrinsic cells into the myofibroblast cells and attenuated the infiltration of macrophages in the progression of renal interstitial fibrosis [30]. Fasudil might also reduce proteinuria by protecting podocyte integrity and decrease the interstitial macrophage density, thereby exerting renoprotective effects against chronic kidney disease [31]. For studying the pharmacological effects of fasudil on glyoxylate-induced nephrolithic injury, Hu et al. [32] utilized a glyoxylate-induced mouse model of kidney calcium oxalate crystal deposition. The study discovered that fasudil reduced the calcium crystal formation and deposition and slowed down the kidney fibrogenesis caused by calcium crystal deposition. And the possible mechanism might be related with the regulatory effects of fasudil on Rho/ROCK signal transduction and EMT.

Fasudil inhibits the activity of ROCK by competing with ATP for the ATP-binding pocket in the active site of ROCK [33]. In 2003, Gu et al. [16] found that 20 μmol/L fasudil could maximally inhibit the transdifferentiation of renal tubular epithelial cells induced by TGF-β1. In our study, fasudil was divided into four groups by concentration (0, 10, 20 and 30 μmol/L) to block renal tubular epithelial cell EMT for 48 h. These experiments demonstrated that, the number of epithelial cells undergoing EMT decreased with increasing fasudil concentrations in groups T2 and T3. The number of epithelial cells undergoing EMT in group T4 were slightly increased compared with group T3, but still significantly less than group T1. This result suggested that the inhibitory effects of fasudil on EMT had reached an apex. This study successfully validated that 20 μmol/L fasudil could inhibit renal tubular epithelial cell transdifferentiation through immunofluorescence, RT-PCR and western blot analyzes. In summary, we conclude that fasudil can reduce the transdifferentiation of renal tubular epithelial cells induced by PTH by inhibiting Rho/ROCK signaling. This discovery provides the experimental basis for using Rho/ROCK inhibitors in the clinical prevention and treatment of renal fibrosis.
